# Modeling vaccination campaigns and the Fall/Winter 2009 activity of the new A(H1N1) influenza in the Northern Hemisphere

**DOI:** 10.3134/ehtj.09.011

**Published:** 2009-11-02

**Authors:** P Bajardi, C Poletto, D Balcan, H Hu, B Goncalves, JJ Ramasco, D Paolotti, N Perra, M Tizzoni, W Van den Broeck, V Colizza, A Vespignani

**Affiliations:** 1 Computational Epidemiology Laboratory, Institute for Scientific Interchange, Turin, Italy; 2 Centre de Physique Théorique, Université d'Aix-Marseille, Marseille, France; 3 Center for Complex Networks and Systems Research, School of Informatics and Computing, Indiana University, Bloomington, IN, USA; 4 Pervasive Technology Institute, Indiana University, Bloomington, IN, USA; 5 Department of Physics, Indiana University, Bloomington, IN, USA; 6 Department of Physics, University of Cagliari, Cagliari, Italy; 7 Linkalab, Cagliari, Italy; 8 Scuola di Dottorato, Politecnico di Torino, Torino, Italy; 9 Lagrange Laboratory, Institute for Scientific Interchange Foundation, Turin, Italy

## Abstract

The unfolding of pandemic influenza A(H1N1) for Fall 2009 in the Northern Hemisphere is still uncertain. Plans for vaccination campaigns and vaccine trials are underway, with the first batches expected to be available early October. Several studies point to the possibility of an anticipated pandemic peak that could undermine the effectiveness of vaccination strategies. Here, we use a structured global epidemic and mobility metapopulation model to assess the effectiveness of massive vaccination campaigns for the Fall/Winter 2009. Mitigation effects are explored depending on the interplay between the predicted pandemic evolution and the expected delivery of vaccines. The model is calibrated using recent estimates on the transmissibility of the new A(H1N1) influenza. Results show that if additional intervention strategies were not used to delay the time of pandemic peak, vaccination may not be able to considerably reduce the cumulative number of cases, even when the mass vaccination campaign is started as early as mid-October. Prioritized vaccination would be crucial in slowing down the pandemic evolution and reducing its burden.

## Introduction

With decreasing trends for pandemic H1N1 cases reported in most of the Southern Hemisphere countries, the concerns regarding the epidemic evolution are now focusing on the influenza activity during Fall 2009 in the Northern Hemisphere.^1–3^ The future unfolding of a pandemic is dominated by a large degree of uncertainty, however, several studies and technical reports recently outlined a likely course of the pandemic in the next few months, identifying plausible scenarios and quantifying the expected impact on the population.^4–8^ The modeling approaches in these studies are characterized by the likelihood of an early epidemic activity in the Northern Hemisphere, with the peak expected to occur in October/November. As an effective line of defense against influenza epidemics, most of the countries are planning the vaccination of a large fraction of the population.^9^ Started after the virus identification at the end^10^ These authors equally contributed to this work. of April 2009, the vaccine development and production is well under way and recently received the approval by the US Food and Drugs Administration.^10^ Vaccine delivery is scheduled to start in early or mid-October^10^ in several countries, but the expected timing of the pandemic influenza activity predicted to peak in October/November puts at risk the effectiveness of mass vaccination as a control strategy.

Here, we use the Global Epidemic and Mobility (GLEaM) model^7, 11^ to assess the effect of mass vaccination on the predicted pandemic evolution, given the expected vaccine availability and timing of distribution. In ref. ^7^, the GLEaM model has been used to perform a Maximum Likelihood Estimate (MLE) of the transmission potential of the current H1N1 pandemic and provide predictions on the unfolding of the current pandemic. Here, we use the model and predicted patterns of global spread obtained in ref. ^7^ to quantify the mitigation effect of mass vaccination campaigns and combined strategies under different scenarios.

## Methods

### Baseline model

To provide pandemic scenarios and test the implementation of mitigation strategies, we use the global epidemic and mobility model (GLEaM), based on a spatially structured meta-population approach^7, 12–23^ in which the world is divided into geographical regions defining a sub-population network where connections among sub-populations represent the individual fluxes because of the transportation and ^4^mobility infrastructures. GLEaM integrates three different data layers:^7, 11^ (i) the population layer at a scale of ^11^ based on the high-resolution population database of the ‘Gridded Population of the World’ project of SEDAC (Columbia University);^24^ (ii) the transportation mobility layer integrating air travel mobility from the International Air Transport Association (IATA)^25^ and OAG^26^ databases, and the commuting patterns and local transportation modes obtained from the data collected and analyzed from more than ^30^ countries in five continents in the world;^7, 11^ (iii) the epidemic layer that defines the disease and population dynamics. The resulting model includes 3362 georeferenced sub-populations centered around major transportation hubs in 220 different countries.^7, 11^

**Figure 1:**
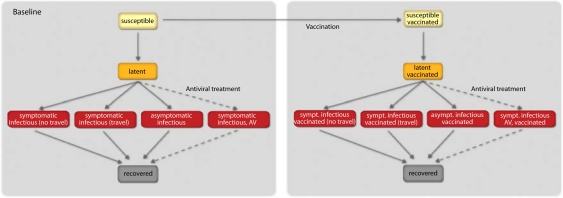
Compartmental structure in each sub-population. A susceptible individual interacting with an infectious person may contract the illness and enter the latent compartment where he is infected, but not yet infectious. At the end of the latency period, each latent individual becomes infectious entering the symptomatic compartment with probability (1-pa) or becoming asymptomatic with probability pa. Asymptomatic individuals infect with a transmission rate reduced of rβ. A fraction (1-pt) of the symptomatic individuals would stop traveling when ill. Infectious individuals recover permanently with rate µ. Antiviral treatment is assumed to be administered to a fraction pAV of the symptomatic infectious individuals within 1 day from the onset of symptoms, according to the drugs availability in the country. It reduces the infectiousness by the antiviral efficacy AVEI and shortens the infectious period of 1 day. If vaccines are available, a fraction equal to 1% of the susceptible population enters the susceptible vaccinated compartment each day. A similar progression to the baseline compartmentalization is considered if infection occurs. However, the vaccine reduces the susceptibility of the vaccinated susceptible with an efficacy VEs, the probability of developing symptoms if infection occurs with an efficacy VED, and their transmission rate while infectious with an efficacy VEi. All transition process are modeled through multinomial processes.

The model simulates short range mobility between sub-populations with a time scale separation approach that defines the effective force of infections in connected sub-populations.^7, 11, 27, 28^ The airline mobility from one subpopulation to another is modeled by an individual based stochastic procedure in which the number of passengers of each compartment traveling from a sub-population *j* to a sub-population *l* is an integer random variable defined by the actual data from the airline transportation database. The infection dynamics takes place within each sub-population and assumes the classic influenza-like-illness compartmentalization in which each individual is classified by one of the following discrete states: susceptible, latent, symptomatic infectious, asymptomatic infectious, permanently recovered/removed.^29, 30^ The model assumes that the latent period is equivalent to the incubation period and that no secondary transmissions occur during the incubation period. All transitions are modeled through binomial and multinomial processes to ensure the discrete and stochastic nature of the processes.^7, 11^ Asymptomatic individuals are considered as a fraction *p*_*a*_=33% of the infectious individuals generated in the model and assumed to infect with a relative infectious ness of *r*_*β*_=50%.^30–32^ Change in traveling behavior after the onset of symptoms is modeled with the probability 1 *p*_*t*_ set to 50% that individuals would stop traveling when ill^30^ (see Figure 1 for a detailed description of the compartmentalization). Effects of variations of these parameters are studied and discussed in the Supplementary Information. In the model, we use values of generation time interval and transmissibility according to the estimates of refs 7, 8. In particular, we use the reproductive number R0=1.75 with the generation interval set to 3.6 days (average latency period of 1.1 days and an average infectious period of 2.5 days).^7^ It is important to remark that the best estimate of the reproductive number refers to the reference value that has to be rescaled by the seasonality scaling function. Seasonality is considered in the model by means of a sinusoidal forcing of the reproductive number, with a scaling factor ranging from amin during Summer season to amax during Winter season.^16^ Here, we consider α_max_=1.1 and α_min_ in the range 0.6–0.7,that is the best estimate obtained from the correlation analysis on the chronology of 93 countries seeded before June 18 in ref. 7 This seasonal scaling provides an effective reproductive number in the Northern hemisphere in the range 1.2–1.6 in the spring/fall months, in agreement with published estimates of the reproductive number. Initial conditions are defined by setting the start of the epidemic near La Gloria in Mexico on 18 February 2009, as in ref. 7 and analogously to other works,^31^ and following available data from official sources.^33^

The above estimates of the seasonal transmission potential is obtained by using the model to perform maximum likelihood analysis of the parameters against the actual chronology of newly infected countries as detailed in ref. 7 The method is computationally intensive as it involves a Monte Carlo generation of the distribution of arrival time of the infection in each country based on the analysis of one million worldwide simulations of the pandemic evolution with the GLEaM model. It is worth stressing that the model assumes homogeneous mixing in each sub-population and full susceptibility. The inclusion of additional structures (such as for example, subdivision in age classes) or agespecific features (such as age-specific transmission) are limited by the lack of data for each of the 220 countries in the world. These assumptions represent a necessary trade-off for the computational efficiency of the model that allows to perform parameter estimations fitting the worldwide pattern of the pandemic,^7^ explore several scenarios under different conditions, and perform sensitivity analysis on the assumptions. Indeed, once the disease parameters and initial conditions are defined, GLEaM generates in-silico epidemics for which we can gather information, such as prevalence, morbidity, number of secondary cases, number of imported cases and many others for each sub-population with a time resolution of one day. All results shown in the following sections are obtained from the statistics based on at least 2000 stochastic runs of the model.

### Intervention strategies

The baseline (no intervention) scenario is studied along with mitigation strategies based on the use of antiviral drugs and the use of vaccines.^12, 18, 30, 32, 34–40^

Intervention involving vaccination is constrained on the availability and distribution of vaccine doses matching the novel H1N1 influenza virus. Current information on the time and amount of delivery of the first doses of vaccine is available for certain countries only and undergoes continuous updates. Significant availability of H1N1 vaccine is expected to begin only in mid-October or later. The United States have initially projected 45 M doses by 15 October, with additional 15 M doses shipped every week after that date, reaching the delivery of the full amount of 195 M doses by the end of December (http://health.usnews.com/articles/ health/healthday/2009/08/21/swine-flu-vaccine-seems-safein-early-trials.html; http://www.pbs.org/newshour/bb/health/july-dec09/h1n1_09-11.html).^41^ The United Kingdom plans to have the first amount of 100,000 doses by mid-October, with subsequent distribution of additional doses till full coverage of the population (http://www.thelancet.com/H1N1-flu/egmn/0c03b3cf). Little is known about vaccine production rates and delivery for several other countries. Here, we assume that all countries having stockpiled on antivirals^42^ would have placed orders to have vaccines available to administer to their populations. On the basis of the available data on vaccination programs, we explore scenarios where the campaign starts on the same date for all countries with vaccines, where the date is set to 15 October,15 November. Additional dates are also studied in the sensitivity analysis. Following previous studies on vaccination during the course of a pandemic,^6, 36, 37^ we assume a dynamic mass vaccination of 1% of the population uniformly in countries where doses are available, till their exhaustion. We assume the administration of a single dose of vaccine,^10, 43, 44^ providing protection with a delay of 2 weeks.^45^ The 2-weeks time to produce the immune response was chosen according to the preliminary data in adult clinical studies for H1N1 influenza vaccine,^10, 45^ and a sensitivity analysis reducing it to 1 week was performed. Recommendations foresee the use of vaccines first in the groups of population who are at elevated risk of severe outcomes or who are likely to come in contact with the novel H1N1 virus.^46^ The model does not consider socialstructure in the sub-populations, therefore the effect of prioritized distribution of vaccines to health care workers, risk groups and others, in reducing the number of hospitalizations and deaths^8, 46–48^ is out of the scope of the present study. Mass vaccination aims to: (i) reduce susceptibility to infection; (ii) reduce infectiousness if infection occurs; (iii) reduce the probability of developing clinical symptoms.^36^ The efficacy of the vaccine with respect of these three effects is quantified by the parameter VES, VEI, VED, respectively. The efficacy of the vaccine is still under study, therefore, we refer to previous estimates and perform a sensitivity analysis to explore higher and lower efficacy levels. Here, we consider a vaccine efficacy for susceptibility VES=70%, a vaccine efficacy for infectiousness VEI=30% and a vaccine efficacy for symptomatic disease given infection VED=50%.^8, 36, 49^ A full description of the disease dynamics in case mass vaccination is considered is available in the Supplementary Information. On the basis of the partial information on total production amounts per country, ranging from ~1/3 of the population^50–52^ to 2/3,41 up to full coverage (http://www. thelancet.com/H1N1-flu/egmn/0c03b3cf),^53, 54^ we explore two different mass vaccination scenarios in which we assume a 30 and a 60% coverage of the population.

We also consider combined strategies including the systematic treatment of clinical cases with antiviral drugs aimed at reducing the severity of the disease and the transmissibility while infectious.^30, 32, 34^ Actual data on antiviral stockpiles in the world are collected from ref. 42 and from national agencies to model the current availability of the drugs by country. We assume the treatment with antivirals of 5 and 10% of clinical cases within the first day from the onset of symptoms, along with a hypothetical conservative intervention with the treatment of 30% of clinical cases. This parameter takes into account the prompt detection of symptomatic cases and the rapid administration of the drug.^7, 12^ The treatment is considered to last until resources are available. We assume a drug efficacy in reducing transmission equal to 62%, and a reduction of 1 day of the total infectious period.^30, 32^ A schematic illustration of the compartmental diagram including the combination of intervention strategies is reported in Figure 1.

## Results and discussion

According to the best estimates of the model parameters as in the previous section, it is possible to calculate the 95% reference range for the activity peak in each country. The benchmark to evaluate the effect of mass vaccination campaigns is the no intervention scenario that is predicted to reach the activity peak for example, in the United States between the beginning of October and the beginning of November. In the following, we will refer to the early and late peak cases as the earliest and latest date, respectively, of the reference range for the activity peak time (see Table 1).^7^ This allows us the consideration of the whole range of peak times to explore the impact of mass vaccination campaigns also in extreme situations such as very early activity peak in October. Although we define the late peak case, it is important to stress that also in this case, we are in the presence of an activity peak occurring much earlier than the usual timing of seasonal influenza. It is also worth remarking that the prediction for the activity peak reference range obtained in the model in the Northern Hemisphere differ from country to country,^7^ as reported in the Table 1 for the countries analyzed here. The model allows the same analysis for 220 countries in the world.

**Table 1:** Relative effect of vaccination in reducing the peak attack rate and the epidemic size with respect to the no intervention scenario

*Vaccination*	*Baseline peak time*	*Relative reduction of peak attack rate (%)*				*Relative reduction of epidemic size (%)*			
			
*Country*		*15 Oc 30% cov*	*15 Oct 60% cov*	*15 Nov 30% cov*	*15 Nov 60% cov*	*15 Oct 30% cov*	*15 Oct 60% cov*	*15 Nov 30% cov*	*15 Nov 60% cov*
US	(23 Sep–09 Nov)	(1–15)	(1–15)	(0–2)	(0–2)	(5–25)	(5–25)	(1–2)	(1–2)
UK	(10 Oct–19 Nov)	(1–29)	(1–29)	0	(0–1)	(11–30)	(11–31)	(1–4)	(1–4)
Canada	(04 Oct–14 Nov)	(1–21)	(1–21)	(0–1)	(0–1)	(10–30)	(10–32)	(1–5)	(1–5)
France	(11 Oct–21 Nov)	(2–32)	(2–32)	(0–2)	(0–2)	(12–32)	(12–33)	(1–5)	(1–5)
Italy	(17 Oct–23 Nov)	(5–38)	(5–38)	(0–1)	(0–1)	(13–35)	(13–36)	(1–5)	(1–5)
Spain	(09 Oct–19 Nov)	(1–30)	(1–30)	(0–1)	(0–1)	(11–32)	(11–33)	(1–4)	(1–4)
Germany	(11 Oct–20 Nov)	(2–34)	(2–34)	(0–1)	(0–1)	(12–33)	(12–34)	(1–4)	(1–4)

Results show the relative reduction obtained with each vaccination strategy with respect to the baseline case. They are calculated as the relative reduction of the maximum of the 95% reference range obtained from 2000 stochastic realizations of the model (vaccination strategy vs baseline), and correspond to the extreme of the reference range for the activity peak time. The 95% reference range of the activity peak in the no intervention scenario is also shown.

**Figure 2:**
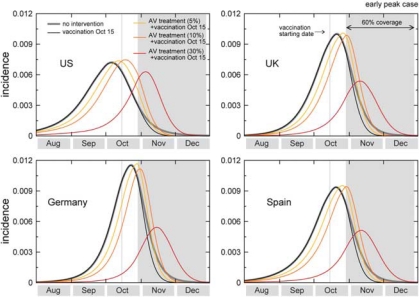
Effect of vaccination and of combined strategies for the early peak case. The incidence curves show the impact of an incremental vaccination with 1% daily distribution policy starting on 15 October for the early peak case. The baseline case is compared with the cases in which intervention strategies are considered, vaccination only, and combination of vaccination with antiviral treatment of 5, 10 and 30% of clinical cases. Efficacies of antiviral treatment and vaccination assume the values reported in the main text. Median profiles obtained from 2000 stochastic realizations of the model are shown. A 60% vaccine coverage is assumed, with the gray bar indicating the time period during which the immunization takes effect.

**Figure 3:**
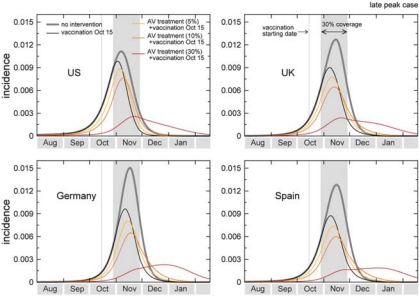
Effect of vaccination and of combined strategies for the late peak case. The incidence curves show the impact of an incremental vaccination with 1% daily distribution policy starting on 15 October for the late peak case. The baseline case is compared with the cases in which intervention strategies are considered, vaccination only, and combination of vaccination with antiviral treatment of 5, 10 and 30% of clinical cases. Efficacies of antiviral treatment and vaccination assume the values reported in the main text. Median profiles obtained from 2000 stochastic realizations of the model are shown. A 30% coverage is assumed, with the gray bar indicating the time period during which the immunization takes effect.

In the case of an activity peak at the beginning of the reference range provided by the model (early October for the United States and many European countries), the mass vaccination program starting on 15 October with 30% coverage would have almost no effect on the epidemic profile, as the effective immunization of incremental 1% of the population would start long after the epidemic has peaked. In the case of a late peak corresponding to the opposite extreme of the reference range (from early to late November depending on the country), the peak attack rate would be reduced by a factor of about 28% averaged across countries, ranging from 15 to 38% depending on the specific pandemic unfolding in each country, with a lower reduction obtained in those countries where the epidemic would arrive earlier (for example, United States vs Europe, according to the predictions of Table 1). Figures 2 and 3 show the incidence curves for a set of countries in the early and late peak cases, respectively. In the United States, for example, the effect of mass vaccination, when no additional intervention strategy is implemented, would correspond to a 15% reduction of the peak incidence in the most favorable situation of a late peak and early vaccination campaign. If the availability of the first vaccine batches is delayed of 1 month, the mass vaccination program would have almost no mitigation effect (<2%) for all countries under study in the whole range of scenarios explored. Moreover, no major differences are observed with a larger coverage, given the 1% daily distribution rate, since in both the early and late peak extreme of the activity peak reference range the assumed 30% coverage would almost always be enough for the distribution during the entire epidemic activity, even assuming the early distribution starting on 15 October. Table 1 summarizes the results for a set of countries, which are expected to deploy vaccination programs in the next Fall, showing expected peak reference ranges and the relative benefit in terms of number of cases of each of the vacination strategies explored at the peak time and at the end of the pandemic wave, with different starts of the campaigns and different coverages. The percentages are calculated as the relative reductions of the maximum of the 95% reference range, where the interval refers to the early and late peak cases (minimum and maximum of the intervals, respectively). According to the above scenarios, the mass vaccination would therefore do little against a pandemic expected to peak before or at the beginning of November, consistently with the simulation results on phased vaccination strategies in the United States.^8^

The introduction of combined mitigation strategies could also help in pushing back the epidemic peak and make more effective the mass vaccination campaigns. Here, we report simulations of scenarios in which the systematic use of antiviral drugs for treatment of cases is used to delay the epidemic peak, and to reduce the attack rate at peak time in combination with the vaccination campaign.^12, 18, 30, 32, 34–40^ If we assume a 5–10% detection of clinical cases and prompt administration of drugs, the pandemic peak is delayed of ~1–2 weeks in the countries with available antiviral stockpiles. We also study a possible scenario of analysis that assumes a 30% treatment, leading to approximately a full month delay of the pandemic peak.^7^ Though larger than the implemented policy for the treatment of clinical cases in some countries, it allows the study of the effectiveness of mass vaccination campaign when a delay of 1 month can be achieved with a combination of intervention strategies.

The delay of 1–2 weeks would allow an additional relative reduction of 10–20% of the peak attack rate with respect to the vaccination only scenario in case of early onset of the mass vaccination campaign. If we consider the early peak case, this would amount to a considerable reduction when compared with the approximately null benefit of the vaccination alone under the same conditions. The results are consistent with those obtained for the case of influenza peaking in the Northern Hemisphere in November and with a mass vaccination campaign starting at the beginning of October in ref. 6. Further mitigation effects would be obtained with a 4 weeks delay because of the antiviral treatment of 30% of the cases. This would allow gaining time for the immunization of a vast percentage of the population to take place. In the early peak situation, the benefit would range from 30 to 59% in reducing the peak attack rate depending on the specific time evolution within each country, and assuming the onset of vaccination on 15 October. In the late peak situation, the mass vaccination would be strongly effective in reducing the attack rate at peak, considerably slowing down the pandemic and mitigating the cumulative number of cases experienced after the first wave. With respect to the maximum reduction of 38% of the peak attack rate in the corresponding vaccination only scenario, a delay of 4 weeks achieved through the combination of mitigation strategies would allow reductions up to 88%, more than doubling the mitigation effect (see the Supplementary Information). This strong mitigation would correspond to a significant benefit in terms of number of cases and in changing the pandemic pattern, thus reducing the burden at peak time on the public health system. Table 2 reports the results obtained for each country when combined strategies with 5 and 10% treatment with antiviral drugs are considered. The results obtained with 30% treatment are reported in the Supplementary Information. The comparison between the results of Table 1 and Table 2 for the same set of assumptions shows that considerably larger mitigation effects would be achieved when combination of different interventions are considered.^18, 36, 38, 40^

**Table 2:** Relative effect of combined strategies in reducing the peak attack rate and the epidemic size with respect to the no intervention scenario

*Combined strategies*	*Relative reduction of peak attack rate (%)*				*Relative reduction of epidemic size (%)*			
		
*Country*	*15 Oct 30% cov*	*15 Oct 60% cov*	*15 Nov 30% cov*	*15 Nov 60% cov*	*15 Oct 30% cov*	*15 Oct 60% cov*	*15 Nov 30% cov*	*15 Nov 60% cov*
*5% AV treatment*								
US	(2–24)	(0–24)	(0–2)	(0–2)	(9–31)	(9–31)	(2–4)	(2–4)
UK	(5–38)	(5–38)	(1–2)	(2–3)	(15–36)	(15–38)	(2–7)	(2–7)
Canada	(1–31)	(1–31)	(0–2)	(0–2)	(14–36)	(14–39)	(2–7)	(2–7)
France	(7–42)	(8–43)	(1–2)	(1–2)	(15–38)	(15–40)	(2–7)	(2–7)
Italy	(11–48)	(11–48)	(1–2)	(1–3)	(17–41)	(17–44)	(2–8)	(2–8)
Spain	(4–41)	(4–41)	(1–2)	(1–2)	(14–38)	(14–40)	(2–7)	(2–7)
Germany	(8–44)	(7–45)	(1–2)	(2–3)	(15–39)	(15–41)	(2–7)	(2–7)
*10% AV treatment*								
US	(1–34)	(2–34)	(1–3)	(1–2)	(13–37)	(13–39)	(3–6)	(3–6)
UK	(12–48)	(12–48)	(4–5)	(3–4)	(19–42)	(19–45)	(4–10)	(4–10)
Canada	(2–42)	(2–42)	(1–3)	(0–1)	(18–42)	(18–48)	(3–10)	(3–11)
France	(14–53)	(14–53)	(3–4)	(3–4)	(20–44)	(20–48)	(4–11)	(4–11)
Italy	(17–58)	(18–58)	(3–4)	(3–4)	(21–46)	(22–52)	(4–12)	(4–12)
Spain	(10–51)	(10–52)	(2–3)	(2–3)	(18–44)	(18–49)	(3–10)	(3–10)
Germany	(14–55)	(14–55)	(3–4)	(3–4)	(19–45)	(19–50)	(4–11)	(4–11)

Results show the relative reduction obtained with each combined strategy with respect to the baseline case, considering the treatment with antivirals to 5 and 10% of clinical cases. The results are calculated as the relative reduction of the maximum of the 95% reference range obtained from 2000 stochastic realizations of the model (combined strategy vs baseline) and at the extreme of the activity peak time reference range reported in Table 1.

Finally, it is worth noting that our model assumes a 100% susceptibility in the population, neglecting effects of previous immunity, since no clear estimates have been provided yet.^55–57^ On the other hand, the global nature of the model allows the simulation of the pandemic since its start in Mexico, taking into account the population-level immunity caused by the first peak of the spread of pandemic H1N1 in the Northern hemisphere during the Spring and Summer 2009. The presented results for the simulated attack rates are likely overestimating the pandemic impact because of the above assumptions. With the best estimate parameters used here, we find clinical attack rates in absence of intervention policies (that is, baseline case) of ~35–40% at the end of the epidemic. A full comparison with attack rates estimates from real data^58^ is, however, made difficult along with the model assumption also by the large underascertainment of cases, the presence of detection biases, surveillance systems with country-specific capacity and coverages, as well as monitoring requirements changing in time as the epidemic progresses. In view of the differences in the outbreak experienced in different countries, we also report in the Supplementary Information the sensitivity analysis on the pandemic transmission potential and generation time. Changes in the effectiveness of the mass vaccination campaign are dependent on the anticipation or delay of the pandemic evolution in the Northern Hemisphere.

### Sensitivity analysis

Although the onset of vaccination is expected for midOctober,^10, 59^ delays could be accumulated in their delivery and administration to the population. A 1 month delay in the start of the vaccination program would preclude the immunization of the public in time for the pandemic wave. If, on the other hand, vaccination programs are put in action starting on 15 October with a larger distribution rate, the mitigation effect would be enhanced. We ran a sensitivity analysis on the 1% incremental vaccination, doubling the vaccine administration rate. Results show a higher mitigation with a variation in the relative reduction of the peak attack rate of about 10% if compared with the corresponding 1% rate, in the case of a 60% vaccine coverage with combination of strategies (see the Supplementary Information).

The preliminary results from the first clinical trials show that a single vaccine dose would produce an immune response in most adults 8–14 days after its administration,^10, 45^ similarly to seasonal influenza vaccines. We tested, therefore, a reduction of the time needed to provide protection, assuming 1 week of time since the administration of the vaccines, with the vaccination onset in mid-October. This is effectively equivalent to a vaccination campaign starting 1 week earlier than 15 October, with the same distribution rate to the public. The anticipation of 1 week—For, equivalently, the faster immunization process after each vaccination—would progressively provide a larger benefit in the mitigation of the pandemic wave, with an additional reduction of about 10% in comparison with the 15 October vaccination onset (see the Supplementary Information). This result confirms that the acceleration of vaccine administration is a key aspect to control next Fall wave.

The efficacy of the H1N1 vaccine is still uncertain. Here, we used as baseline values of efficacy the ones estimated for seasonal influenza,^8, 36, 49^ and explored a vaccine efficacy for susceptibility in the range (50–90%), along with a larger vaccine efficacy for infectiousness, equal to 80%.^37^ The resulting effects in the mitigation of the peak attack rate are limited to variations of up to 5% with respect to the baseline values of the efficacies, showing that the timing and distribution rates have a larger role in the mitigation with respect to the above variations in the efficacies. All results of the analysis are reported in the Supplementary Information.

## Conclusions

The interplay between the timing of the pandemic and the start of the dynamic vaccination campaign is crucial for mitigation effects. Results show that mass vaccination may have little effect on controlling the pandemic even when administered as early as mid-October, unless additional mitigation strategies are considered to delay the activity peak. This makes also a strong case for prioritized vaccination programs focusing on high-risk groups, healthcare and social infrastructure workers. Should the pandemic peak much later than anticipated from the modeling approach, in December or January, there would be enough time to provide immunization to a larger fraction of the population given the current schedule for vaccination campaign, with a larger mitigation effect than in the early pandemic wave situation.

## Notes

Supplementary Information accompanies the paper on Emerging Health Threats Journal website (http://www.eht-journal.org)
